# Supplementary material for: Oral Treatment with RD2RD2 Impedes Development of Motoric Phenotype and Delays Symptom Onset in SOD1G93A Transgenic Mice
Source: Int J Mol Sci. 2021 Jun 30;22(13):7066. doi: 10.3390/ijms22137066 (PMC8269060; doi:10.3390/ijms22137066)
Supplement: Supplementary file 1 [file ijms-22-07066-s001.zip › ijms-1269496-supplementary.pdf]

Supplement

Oral treatment with RD2RD2 impedes development of motoric phenotype and delays symptom onset in $SOD1^{G93A}$ transgenic mice

Julia Post[1], Anja Schaffrath[1], Ian Gering[1], Sonja Hartwig[2,3], Stefan Lehr[2,3], N. Jon Shah[4,5,6,7], Karl-Josef Langen[4,8], Dieter Willbold[1,9]*, Janine Kutzsche[1]*, Antje Willuweit[4]*

Affiliations

[1]Institute of Biological Information Processing, Structural Biochemistry (IBI-7), Forschungszentrum Jülich, Jülich, Germany

[2]Institute for Clinical Biochemistry and Pathobiochemistry, German Diabetes Center, Leibniz Center for Diabetes Research at Heinrich-Heine-University Düsseldorf, 40225 Düsseldorf, Germany

[3]German Center for Diabetes Research, Partner Düsseldorf 85764 München-Neuherberg, Germany

[4]Institute of Neuroscience and Medicine, Medical Imaging Physics (INM-4), Forschungszentrum Jülich, Jülich, Germany

[5]Institute of Neuroscience and Medicine 11, INM-11, JARA, Forschungszentrum Jülich, Jülich, Germany

[6]JARA - Brain - Translational Medicine, Aachen, Germany

[7]Department of Neurology, RWTH Aachen University, Aachen, Germany

[8]Department of Nuclear Medicine, RWTH Aachen University, Aachen, Germany

[9]Institut für Physikalische Biologie, Heinrich-Heine-Universität Düsseldorf, Düsseldorf, Germany

* Correspondence to:

Antje Willuweit

Forschungszentrum Jülich GmbH
Institute of Neuroscience and Medicine, Medical Imaging Physics (INM-4)
52425 Jülich, Germany
e-mail: a.willuweit@fz-juelich.de
phone: +49-2461-6196358
fax: +49-2461-612302
and

Janine Kutzsche

Forschungszentrum Jülich GmbH
Institute of Biological Information Processing, Structural Biochemistry (IBI-7)
52425 Jülich, Germany
e-mail: j.kutzsche@fz-juelich.de
phone: +49-2461-619496
fax: +49-2461-619497
and

Dieter Willbold

Forschungszentrum Jülich GmbH
Institute of Biological Information Processing, Structural Biochemistry (IBI-7)
52425 Jülich, Germany
e-mail: d.willbold@fz-juelich.de
phone: +49-2461-612100
fax: +49-2461-612023

Methods
Stability measurement of RD2RD2

RD2RD2 was formulated in tailor-made jellies as described in the method section of the manuscript. The concentration of RD2RD2 was determined in freshly made (day 0) and 6 day-old jellies by reversed-phase high-performance liquid chromatography (RP-HPLC). A concentration of RD2RD2 was measured depending on the dosage of 50 mg/kg and thus on the weight of the $SOD1^{G93A}$ mice (14 week-old transgenic mice, average weight: 18.0 g, RD2RD2: 1414 µM). Prior to the measurement RD2RD2 was extracted from the jellies by addition of 200 µL of 20 % (*w/v*) trichloroacetic acid (TCA) (Roth, Karlsruhe, Germany). After resuspension and centrifugation at 14.000 x g for 10 min at 4 °C, the supernatant was analysed by RP-HPLC using the Agilent 1260 Infinity II system (Agilent Technologies, Santa Clara, USA). Chromatography was performed on a C18 column (Agilent Technologies, Zorbax 300SB-C18 5 µm, 4.6 mm x 250 mm; Santa Clara, USA) at 25 °C with a flow rate of 1 mL/min. Ultraviolet absorbance was recorded at 214 nm. The sample injection volume was 20 µL. Chromatograms were recorded and analysed by Agilent OpenLAB CDS (software version 2.5, Agilent Technologies, Santa Clara, USA). For sample analysis from stability tests, mobile phases consisted of water (A) and acetonitrile (B) each supplemented with 0.1 % (*v/v*) TCA (AppliChem, Darmstadt, Germany). The samples were measured isocratically at 10 % solvent B for 10 min. Afterward, a gradient was applied from 10 % to 45 % of the acetonitrile solvent in a measurement of 20 min. The samples were measured in triplicate and normalised peak areas of each measurement were averaged. GraphPad Prism 8 (GraphPad Software Inc., USA) was used for the graphic illustrations. Data are presented as mean ± SEM.

Figures

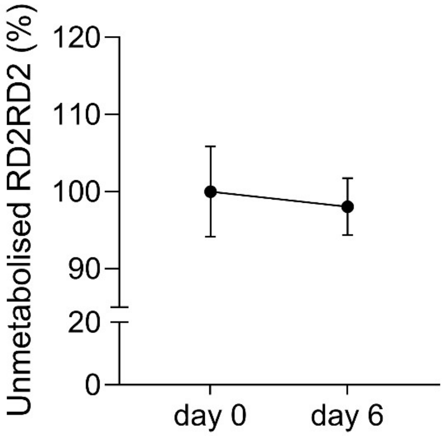

Figure S1: Stability of RD2RD2 in tailor-made jellies. RD2RD2 concentration was determined in freshly made (day 0) and 6 days-old. RP-HPLC measurement revealed a minimum loss of RD2RD2 after 6 days of incubation (1.95 ± 3.69 %), however RD2RD2 remained stable. Data are presented as mean ± SEM (n = 3).

Tables

Table S1: Analysis of ALS pathology in brain and lumbar spinal cord sections of RD2RD2-treated $SOD1^{G93A}$ mice and non-transgenic littermates. Data indicate a significant change in neurodegeneration (staining with NeuN and ChAT antibodies) and neuroinflammation (staining with antibodies against CD11b and GFAP) upon 63 days of *per oral* treatment with RD2RD2. Data are represented as mean ± SEM. Statistical calculations were conducted by one-way ANOVA with Fisher's LSD post hoc analysis. Asterisks (*) indicate significance between non-transgenic and transgenic treatment groups (ntg vs placebo or ntg vs RD2RD2: * p = 0.05, ** p = 0.001 and *** p < 0.001). Lozenges ($^{\#}$) indicate significance between transgenic treatment groups (placebo vs RD2RD2: $^{\#}$ p = 0.05, $^{\#\#}$ p = 0.01 and $^{\#\#\#}$ p < 0.001). p values of > 0.05 was considered to be statistically not significant (ns). IR: immunoreactivity

IR (%)	area	ntg	placebo	RD2RD2	statistic
CD11b	brain stem	2.28 ± 0.34	5.34 ± 0.43 ***	3.86 ± 0.39 **, $^{\#}$	F(2,39) = 15.6, p < 0.001<br>ntg vs placebo p < 0.001<br>ntg vs RD2RD2 p = 0.006<br>placebo vs RD2RD2 p = 0.010
	lumbar spinal cord	1.58 ± 0.34	5.18 ± 0.91 ***	4.26 ± 0.65 **	F(2,26) = 8.14, p = 0.002<br>ntg vs placebo p < 0.001<br>ntg vs RD2RD2 p = 0.007<br>placebo vs RD2RD2 ns
GFAP	brain stem	2.42 ± 0.39	6.75 ± 0.59 ***	4.24 ± 0.46 *,$^{\#\#\#}$	F(2,36) = 19.1, p < 0.001<br>ntg vs placebo p < 0.001<br>ntg vs RD2RD2 p = 0.016<br>placebo vs RD2RD2 p < 0.001
	lumbar spinal cord	3.23 ± 0.78	7.69 ± 1.39 *	8.55 ± 1.28 **	F(2,27) = 5.88, p = 0.008<br>ntg vs placebo p = 0.012<br>ntg vs RD2RD2 p = 0.004<br>placebo vs RD2RD2 ns
**counts**	**area**	**ntg**	**placebo**	**RD2RD2**	**statistic**
NeuN	brain stem	316 ± 29.9	182 ± 16.4 ***	273 ± 27.1 $^{\#\#}$	F(2,35) = 7.68, p = 0.002<br>ntg vs placebo p < 0.001<br>ntg vs RD2RD2 ns<br>placebo vs RD2RD2 p = 0.009
	motor cortex	641 ± 33.8	509 ± 32.1 **	605 ± 23.6 $^{\#}$	F(2,26) = 5.08, p = 0.014<br>ntg vs placebo p = 0.005<br>ntg vs RD2RD2 ns<br>placebo vs RD2RD2 p = 0.038
ChAT	brain stem	564 ± 45.2	402 ± 30.1 **	477 ± 27.3	F(2,30) = 5.32, p = 0.010<br>ntg vs placebo p = 0.003<br>ntg vs RD2RD2 ns<br>placebo vs RD2RD2 ns

Table S2: Pearson's correlation coefficient between SHIRPA scores and cell quantifications. Asterisks (*) indicate significance between all three treatment groups (overall effect: ntg vs placebo vs RD2RD2: * p = 0.05, ** p = 0.01 and *** p < 0.001). r = Pearson correlation coefficient, IR: immunoreactivity

<table>
<tr><th colspan="7">SHIRPA score vs</th></tr>
<tr><td>area</td><td>CD11b (IR %)</td><td>GFAP (IR %)</td><td>area</td><td>NeuN (counts)</td><td>area</td><td>ChAT (counts)</td></tr>
<tr><td>brain stem</td><td>r = 0.32 *</td><td>r = 0.67 ***</td><td>brain stem</td><td>r = - 0.51 ***</td><td rowspan="2">brain stem</td><td rowspan="2">r = - 0.46 **</td></tr>
<tr><td>lumbar spinal cord</td><td>r = 0.49 **</td><td>r = 0.46 **</td><td>motor cortex</td><td>r = - 0.47 *</td></tr>
<tr><th colspan="7">SHIRPA motor score vs</th></tr>
<tr><td>area</td><td>CD11b (IR %)</td><td>GFAP (IR %)</td><td>area</td><td>NeuN (counts)</td><td>area</td><td>ChAT (counts)</td></tr>
<tr><td>brain stem</td><td>r = 0.72 ***</td><td>r = 0.65 ***</td><td>brain stem</td><td>r = - 0.52 ***</td><td rowspan="2">brain stem</td><td rowspan="2">r = - 0.51 **</td></tr>
<tr><td>lumbar spinal cord</td><td>r = 0.66 ***</td><td>r = 0.45 *</td><td>motor cortex</td><td>r = - 0.44 *</td></tr>
</table>

Table S3: Analysis of plasma revealed differences in several cytokine levels of SOD1^{G93A} mice vs non-transgenic littermates and between RD2RD2 and placebo-treated mice. Cytokine levels were determined using a multiplex immunoassay. Cytokine concentrations are given in picogram per millilitre (pg/mL). Data is represented as mean ± SEM. Statistical calculations were conducted by one-way ANOVA with Fisher's LSD post hoc analysis, ntg n = 7, placebo n = 11 and RD2RD2 n = 11. Asterisks (*) indicate significance between non-transgenic and transgenic treatment groups (ntg vs placebo and ntg vs RD2RD2: * p = 0.05, ** p = 0.01 and *** p < 0.001). Lozenges ($^{\#}$) indicate significance between transgenic treatment groups (placebo vs RD2RD2: $^{\#}$ p = 0.05).

concentration (pg/mL)	ntg	placebo	RD2RD2	statistic
IL-1β	797 ± 33.1	545 ± 97.0 *	685 ± 70.2	$F(2,22) = 2.61$, $p = 0.096$<br>ntg vs placebo $p = 0.033$<br>ntg vs RD2RD2 ns<br>placebo vs RD2RD2 ns
IL-4	28.3 ± 2.45	25.6 ± 3.46	23.8 ± 2.81	$F(2,18) = 0.68$, $p = 0.516$<br>ntg vs placebo ns<br>ntg vs RD2RD2 ns<br>placebo vs RD2RD2 ns
IL-6	63.6 ± 2.94	43.9 ± 6.29 *	57.1 ± 6.99	$F(2,21) = 2.62$, $p = 0.096$<br>ntg vs placebo $p = 0.037$<br>ntg vs RD2RD2 ns<br>placebo vs RD2RD2 ns
IL-10	254 ± 9.55	159 ± 15.4 **	217 ± 23.6 [#]	$F(2,21) = 6.33$, $p < 0.007$<br>ntg vs placebo $p = 0.002$<br>ntg vs RD2RD2 ns<br>placebo vs RD2RD2 $p = 0.033$
IL-12p40	344 ± 22.6	554 ± 51.4 *	533 ± 63.5 *	$F(2,26) = 3.64$, $p = 0.040$<br>ntg vs placebo $p = 0.017$<br>ntg vs RD2RD2 $p = 0.031$<br>placebo vs RD2RD2 ns
IL-13	1028 ± 59.0	546 ± 64.1 ***	748 ± 73.2 *, [#]	$F(2,24) = 11.48$, $p < 0.001$<br>ntg vs placebo $p < 0.001$<br>ntg vs RD2RD2 $p = 0.011$<br>placebo vs RD2RD2 $p = 0.036$
IL-17	214 ± 25.1	129 ± 20.1 *	130 ± 26.8 *	$F(2,23) = 4.66$, $p = 0.020$<br>ntg vs placebo $p = 0.007$<br>ntg vs RD2RD2 $p = 0.027$<br>placebo vs RD2RD2 ns
INF-γ	189 ± 10.4	190 ± 23.5	173 ± 23.7	$F(2,21) = 0.21$, $p = 0.816$<br>ntg vs placebo ns<br>ntg vs RD2RD2 ns<br>placebo vs RD2RD2 ns
TNF-α	1456 ± 105	1095 ± 155	1363 ± 174	$F(2,22) = 1.45$, $p = 0.255$<br>ntg vs placebo ns<br>ntg vs RD2RD2 ns<br>placebo vs RD2RD2 ns
CCL-2	699 ± 48.4	444 ± 55.8 **	612 ± 55.4 [#]	$F(2,23) = 5.25$, $p = 0.013$<br>ntg vs placebo $p = 0.005$<br>ntg vs RD2RD2 ns<br>placebo vs RD2RD2 $p = 0.034$
CCL-5	48.6 ± 4.47	43.6 ± 4.36	45.3 ± 3.94	$F(2,24) = 0.30$, $p = 0.740$<br>ntg vs placebo ns<br>ntg vs RD2RD2 ns<br>placebo vs RD2RD2 ns
CXCL-1	73.2 ± 4.37	123 ± 11.7 **	114 ± 11.4 *	$F(2,23) = 5.63$, $p = 0.010$<br>ntg vs placebo $p = 0.004$<br>ntg vs RD2RD2 $p = 0.013$<br>placebo vs RD2RD2 ns
